# Influence of Nanofiber Orientation on Morphological and Mechanical Properties of Electrospun Chitosan Mats

**DOI:** 10.1155/2018/3651480

**Published:** 2018-11-13

**Authors:** Paola Nitti, Nunzia Gallo, Lara Natta, Francesca Scalera, Barbara Palazzo, Alessandro Sannino, Francesca Gervaso

**Affiliations:** ^1^Department of Engineering for Innovation, University of Salento, Lecce 73100, Italy; ^2^Ghimas S.p.A. c/o Dhitech Scarl, Lecce 73100, Italy

## Abstract

This work explored the use of chitosan (Cs) and poly(ethylene oxide) (PEO) blends for the fabrication of electrospun fiber-orientated meshes potentially suitable for engineering fiber-reinforced soft tissues such as tendons, ligaments, or meniscus. To mimic the fiber alignment present in native tissue, the CS/PEO blend solution was electrospun using a traditional static plate, a rotating drum collector, and a rotating disk collector to get, respectively, random, parallel, circumferential-oriented fibers. The effects of the different orientations (parallel or circumferential) and high-speed rotating collector influenced fiber morphology, leading to a reduction in nanofiber diameters and an improvement in mechanical properties.

## 1. Introduction

In the recent years, tissue engineering (TE) has emerged as an alternative approach to classical surgery of tissues and organs. In TE approach, a three-dimensional (3D) support, namely, the scaffold, is used to allow cell migration, proliferation, and differentiation, and hence repairing damaged tissues [1]. One of the major goals of TE is the design of scaffolds with optimized surface properties for cell interactions and mimicking as better as possible the microarchitecture of native extracellular matrix (ECM) surrounding the cells in the microenvironment [2, 3]. The ECM components, mainly proteoglycans and protein, are arranged as nano/microfibers (50–500 nm diameter), allowing the formation of highly interconnected porous network with the adequate structural resilience for specific cellular function [4, 5]. Recently, the size and topographical features of ECM structural elements have been identified as a key characteristic that can direct cell behavior [2, 6]. In this context, not only the biomaterial composition but also the processing technology plays a key role in the realization of scaffolds with a high degree of biomimicry. To this aim, polymeric nanofibers could be a good choice to mimic structure and function of natural ECM and, among the various techniques available for nanofiber fabrication, electrospinning (ES) is very promising due to its simplicity, environmentally friendly nature, cost-effectiveness, and scalability [7]. ES allows to produce membranes with controllable fiber diameter, alignment, and composition using a variety of synthetic or natural polymers. A significant number of natural tissues exhibit a preferred fiber alignment that gives tissue unique functional properties (e.g., parallel collagen fibers in tendon and ligament or circumferential direction fibers in meniscus) [8]. Such natural fiber-reinforced tissues, as tendons, ligaments, and meniscus, are characterized by a structural anisotropy that governs their mechanical properties and is strictly related to their function. Many studies have shown that the fibers orientation of the substratum influences cell adhesion and growth [9–11] and modulates the elongated cellular patterns that are typical of native tissues [12, 13]. Scaffolds with topography similar to native tissue have the ability to direct the alignment of cells and subcellular structures and successively allow the deposition of collagen along electrospun nanofiber direction that will result in an increase of the tensile properties of the new tissue [14]. To address this issue, mats with controlled fiber alignment can be produced by directing fiber deposition onto opportunely designed collectors. In general, a randomly oriented network of fibers is collected on static targets, whereas aligned fibers are collected on rotating collectors such as drums or disks [15]. The biomimicry of scaffolds is influenced also by biomaterial composition and biodegradation properties. Indeed, polymer biodegradation, driven by the combined effect of enzymatic and hydrolytic activities, generates space within the scaffold that enables cellular processes, such as proliferation and deposition of newly synthesized ECM [15]. The need of high tissue biocompatibility and biodegradation has favored the use of natural polymers like collagen, hyaluronic acid, silk, and chitosan [16]. Among various biopolymers used, chitosan (Cs) (cationic biopolymer obtain by partial de-*N*-deacetylation of chitin) has a great potential because of its biocompatibility, biodegradability, nonantigenicity, antimicrobial activity, and low cost and easy availability [17, 18], especially in comparison with collagen, of which could be considered a valid alternative. Various researchers tried to process Cs into nanofiber mats in order to mimic native ECM, but its low solubility, high viscosity, rigid crystalline structure, and hydro-colloid nature represented a strong limitation in Cs usage [19]. Pure chitosan has been processed by dissolution in concentrated acetic acid (AA) [20] or trifluoroacetic acid (TFA) [21], but, unfortunately, these solvents, especially TFA, are not suitable for biomedical applications because they are hardly removable and consequently toxic. Furthermore, these mats showed inferior mechanical properties and very high swelling ratios [22, 23]. In order to overcome the difficulties of Cs electrospinning, Cs nanofibers have been spun in blend with cospinning polymers that facilitate entanglement of the polymer molecules, while reducing the solution viscosity necessary for fiber formation. Biopolymers used in blend with Cs are poly(ethylene oxide) (PEO), poly[(l-lactide)-co-(d, l-lactide)] (PLA), poly(vinylalcohol) (PVA), and poly(vinyl pyrrolidone) (PVP). PEO is particularly suitable for blending because of its low toxicity, excellent electrospinnability, hydrophilicity, and biocompatibility. Flexible PEO molecules in Cs-PEO blend promote solution flowability along rigid chitosan chains providing needed entanglement for electrospinning [24]. Moreover, PEO can be easily washed out from electrospun Cs fibers by water [22]. Therefore, with the intent of obtaining highly biomimetic matrices for the regeneration of highly oriented soft tissues, the present study aimed (i) to electrospun Cs mats in blend with PEO, with different preferred fiber orientations by using different collector geometries, and (ii) to assess the influence of such orientations on the morphological and mechanical properties of the electrospun mats. Fiber alignment have been addressed by controlling the motion of the collecting mandrel with respect to the electrospinning suspension source [25]. Three types of collector have been used: (1) static plate collector, (2) drum rotating collector, and (3) disk rotating collector, to produce, respectively, random fibers, one direction-orientated fibers, and circumferentially orientated fibers. Notably, the circumferential patterning is necessary for load transmission across the knee joint and, therefore, can be considered a key design parameter for engineering specific tissue constructs, such as meniscus. However, to the best of our knowledge, no studies evaluated the feasibility of spinning Cs mats on a rotating disk as collector to get circumferentially orientated fibers. The effect of this orientation has hence been here studied and compared with fibers deposited using the other typologies of collectors.

## 2. Materials and Methods

### 2.1. Materials

Chitosan (Cs) from shrimp shells low viscosity, poly(ethylene oxide) (Mw 900 and 400 kDa) (PEO), and acetic acid were used as received from Sigma Aldrich, MO, USA. All aqueous solutions were prepared with deionized and Milli-Q water.

### 2.2. Preparation of the Solution for Electrospinning

Cs and PEO powders were mixed together in weight ratio of 70/30 and then dissolved in aqueous acetic acid (90 v/v%) to form 4.5 w/v% Cs/PEO solution. The blend solution was prepared under magnetic stirring overnight at room temperature to obtain homogeneous solution.

### 2.3. Electrospinning of Chitosan Nanofibers

**Figure 1:**
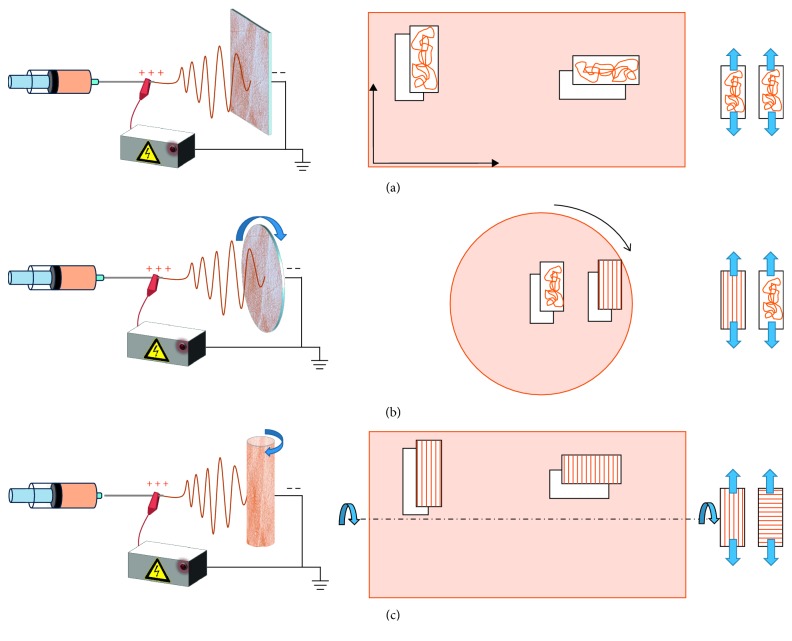
Electrospinning setup. (a) Static collector, (b) disk collector, and (c) drum collector.

The electrospinning system used for fiber preparation consisted of a syringe pump (Model KDS-200-CE), a high-voltage DC power supply generator (model QCHV-M40, Linari Engineering s.r.l., Italy), a static plate collector (Figure 1(a)), a disk collector ((diameter 100 mm) (Figure 1(b)), and a cylindrical rotating drum (30 mm diameter, 120 mm length) with a controllable rotating speed from 0 to 3000 rpm (model RT-Collector Web, Linari Engineering s.r.l, Italy) (Figure 1(c)).

The blend solution was placed into a 20 mL plastic syringe equipped with a metallic needle, of 52 mm length and 0.8 mm inner diameter. The metallic needle was connected to the positive electrode of the high-voltage DC power supply, while the collector was connected to the negative electrode. The spinneret was directed perpendicularly to collector that was wrapped with an aluminum foil. The needle to collector distance was set to 13 cm. The syringe pump released polymer solution at a flow rate of 0.02 ml/min. A 20–22 kV electric field was maintained between the two electrodes. For the disk collector, the rotating speed was varied from 800 to 1200 rpm (800–1000–1200 rpm), while for the drum collector from 800 to 2500 rpm (800–1000–1200–1800–2000–2500 rpm), and the translation speed was fixed at 10 mm/s (Table 1). All experiments were carried out at a room temperature of 25°C. Cs/PEO mats were then peeled off from the collector and dried under vacuum at 50°C for 17 h prior to any further use.

### 2.4. Scanning Electron Microscopy

Scanning electron microscopy (SEM EVO® 40, Carl Zeiss AG) using variable pressure mode and an accelerating voltage of 20 kV was used to observe the surface morphology of chitosan nanofiber mats. Before microscopy analysis, small discs (10 mm diameter) were punched out from each electrospun mat, placed onto the sample holder, and sputter-coated with gold (7 nm) in a vacuum chamber.

SEM micrographs were then processed and analyzed with ImageJ 1.50c. software (NIH, http://rsb.info.nih.gov/ij) to determine the average fibers diameter and size distribution in the electrospun samples (with diameter *ϕ* in the range <100 nm, 100 < *ϕ* < 250 nm, 250 < *ϕ* < 400 nm, 400 < *ϕ* < 550 nm, and 550 < *ϕ* < 800 nm), by taking the average values from 200 measurements chosen randomly in the images of each sample. The diameters are reported as average value ± standard deviation.

### 2.5. Fiber Alignment Analysis via FFT

In order to evaluate the relative fiber alignment of electrospun membranes, FFT (*fast Fourier transform*) analysis of mats SEM images (magnification 2000X) was performed [26]. The FFT function converts the information contained in an optical data image from a “real” domain into a mathematically defined “frequency” domain [27]. In the output image of the FFT analysis (frequency plot), grayscale pixels are distributed in a pattern that reflects the degree of fiber alignment of the original data image [28]. The analysis is based on the principle that a fiber will produce a sharp intensity peak in the frequency domain, which is perpendicular to the fiber direction. All images were stored and analyzed as uncompressed TIF files. Grayscale 8-bit images were cropped to 2048 × 2048 pixels for analysis. The FFT frequency distribution was obtained using ImageJ software supported by an oval profile plug-in (authored by William O'Connnell). an oval projection was placed on the frequency plot and a radial summing of the pixel intensities for each angle between 0° and 360° in oval projection [25]. These values were shifted by 90° to match the orientation of the original images. All alignment data were normalized and plotted in arbitrary units ranging from 0 to approximately 0.15. Using circular statistics, the mean angle of the distribution was calculated for each image. The position of the peak on the plot reports the principal axis of alignment. The 2D FFT alignment plot of a random matrix exhibits four peaks that occur every 90°. Peaks of nearly uniform height are a hallmark of a scaffold composed of random elements. These peaks reflect low-frequency spatial information, and the 2D FFT alignment value is <0.05 units. On the contrary, the FFT plot having two sharp peaks at a distance of 180° is typical of orientated structures. The 2D FFT alignment plots generated from scaffolds containing aligned elements exhibit asymmetrical peaks, two larger peaks at 90° and 270° and two smaller peaks at 0° and 360°. The degree of alignment present in the original data image is reflected by the height and overall shape of the most prominent peaks present in the alignment plot. A high and narrow peak indicates a more uniform degree of fiber alignment, while a broad peak or a shoulder on the peak indicates that more than one axis of alignment may be present. When 2D FFT alignment value is >0.065 units, scaffolds with aligned fibers have been obtained [29].

This 2D FFT analysis was assessed on SEM images of samples produced with static plate and drum collector (800, 1000, 1200, 1800, 2000, and 2500 rpm speed rotating collector).

### 2.6. PEO Removal

In order to evaluate the total PEO removal from the chitosan fibers, the percentage weight loss of samples was calculated and morphological observations were performed before and after immersion in PBS. Firstly, the electrospun Cs/PEO mats were soaked in PBS 1X pH 7.4 for 15, 30, 60, 120, and 300 min. Afterwards, the mats were rinsed with Milli-Q water. The produced fibrous mats were then freeze-dried overnight to remove the absorbed water. The percentage weight loss of samples after immersion was then calculated using the following equation:

(1) $$\begin{array}{c} \%\text{PEO loss}=\left(1-\frac{W_{\text{f}}}{W_{\text{i}}}\right)\times 100, \end{array}$$

where *W*_i_ is the initial sample weight and *W*_f_ is the final weight after immersion. At each time interval, six specimens for each kind of material were tested. Additionally, a SEM analysis was performed to estimate the average fiber diameter and size distribution in the electrospun samples before and after soaking in PBS.

Further analysis using differential scanning calorimetry (DSC, TA Instruments) was made to confirm the total PEO removal. Samples (3 mg, *n*=3) were placed in aluminum pans and heated from 25°C to 300°C at 10°C/min under a 50 ml/min nitrogen flow, using an empty pan as a reference. Raw chitosan and PEO powders were also analyzed and compared with mats before and after immersion in PBS.

### 2.7. Mechanical Properties

The tensile mechanical properties of chitosan mats produced with static, disc, and drum collector at different spindle rotations (800–1000–1200 rpm and 800–1000–1200–1800–2000–2500 rpm for disk and drum respectively) were evaluated in wet condition using a universal testing machine (Zwick Roell, Germany) equipped with a 100 N load cell at a displacement rate of 0.1 mm/s and with a preload of 0.1 N.

Samples for mechanical testing were cut out from each mat in a rectangular shape of 30 mm × 10 mm and immersed in PBS 1X at room temperature for 2 h, i.e., the time needed to totally remove PEO and to reach a complete hydration. The thickness, length, and width of wet rectangular specimens were measured using Stereomicroscopes Nikon SMZ1270 equipped with software image analysis Niss elements AR460. Samples were tested under displacement control till failure along the parallel and perpendicular orientations assuming that, for the rotating collectors, the parallel orientation is in the direction of disk and mandrel rotation (Figure 1). For the static collector, samples along vertical and horizontal directions of the electrospun membranes were considered as parallel and perpendicular, respectively. Six samples from each group were tested and the average Young's modulus (E) was calculated as the slope of the linear elastic region of the stress-strain curve at low strain values (in the range 0–5%). The results were expressed as average value ±standard deviation. Data sets were screened by Student's *t*-test (*p* < 0.05) to assess the effects of mandrel rotation speed on material properties.

## 3. Results

### 3.1. Evaluation of PEO Removal

**Figure 2:**
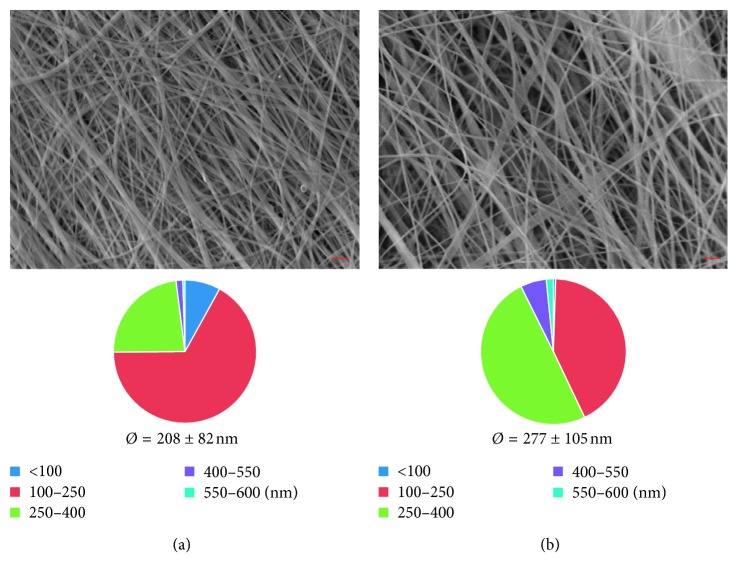
SEM micrographs (scale bar: 2 *µ*m), fiber size distribution, and average diameters of Cs/PEO mat produced at 2000 RPM before (a) and after (b) hydration.

In this study, chitosan/PEO nanofibrous mats with different fiber orientations were produced and a certain amount of fiber-forming additives like PEO was used to improve the Cs electrospinnability. However, PEO is soluble in aqueous medium and its elimination was intended; hence, PEO removal was evaluated. In particular, preliminary tests were performed to assess the time needed to entirely remove the water-soluble PEO by weighting membrane samples before and after PBS immersion (15, 30, 60, 120, and 300 min). The results indicated that after two hours, the membrane weight decreases by 28%, corresponding to the initial PEO amount. The morphological studies (Figure 2) show how the fibers in the swollen state allowed retaining the morphology of the hydrated state. However, the diameter distribution analysis showed how the simultaneous PEO loss and water entry cause fiber swelling and a consequent shift of average diameters from 100–250 nm to 250–400 nm.

**Figure 3:**
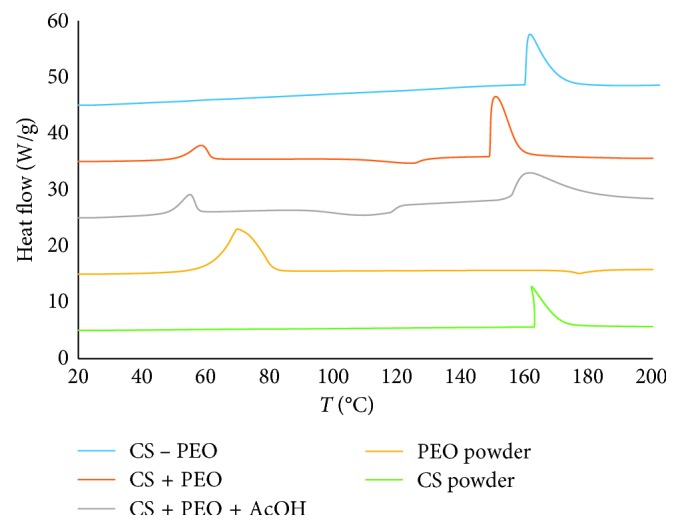
DSC curves of Cs and PEO powder and mats before (Cs + PEO) and after (Cs − PEO) hydration.

The PEO removal was confirmed by DSC analysis (Figure 3). Indeed, DSC curves of mats after immersion in PBS showed the absence of typical PEO peak at 68°C.

### 3.2. Chitosan Random Nanofiber Mats: Static Plate Collector

**Figure 4:**
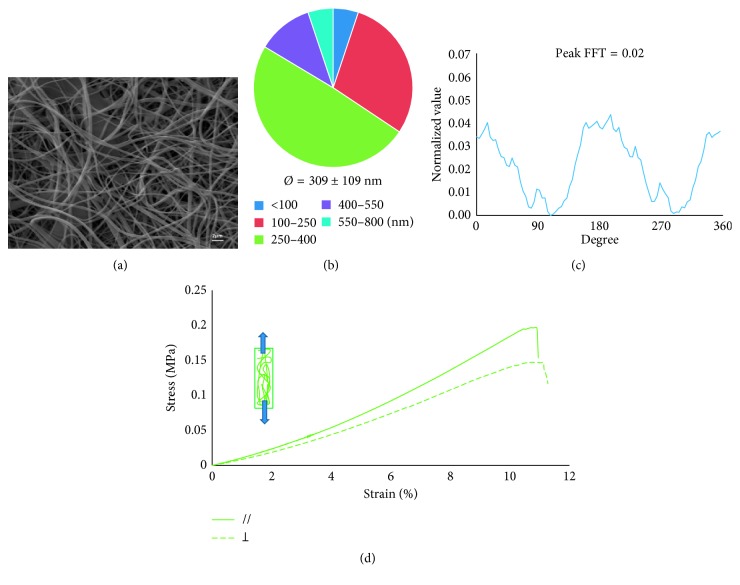
Cs/PEO produced with the static collector. (a) SEM image (scale bar 2 *µ*m), (b) fiber size distribution and average diameters calculated using Image J software, (c) 2D FFT analysis, and (d) stress-strain curve.

A static plate collector was used to electrospin nanofiber mats with random orientation (Figure 1(a)). An example of a random Cs/PEO mat is shown in Figure 4(a). The random mats presented an average fiber diameter of 300 ± 100 nm and most of the fiber diameter (about 50%) followed in the 250–400 nm range (Figure 4(b)). Furthermore, FFT analysis (Figure 4(c)) confirmed the absence of any preferred orientation, as deduced from the low value of the 2D FFT alignment peaks (90° and 270°), that is, ∼0.02.

The mechanical properties of the random-oriented membranes are shown in Figure 4(d). The stress-strain curves of two samples, tested in parallel and perpendicular direction of fibers orientation, are very similar each other and present a slightly nonlinear elastic behavior till failure. For both typologies of specimens, Young's modulus was equal to 1.52 ± 0.29 MPa, whereas stress and elongation at break were, respectively, 0.18 ± 0.04 MPa and 10.81 ± 1.61%.

### 3.3. Chitosan Parallel-Orientated Nanofiber Mats: Rotating Drum Collector

**Figure 5:**
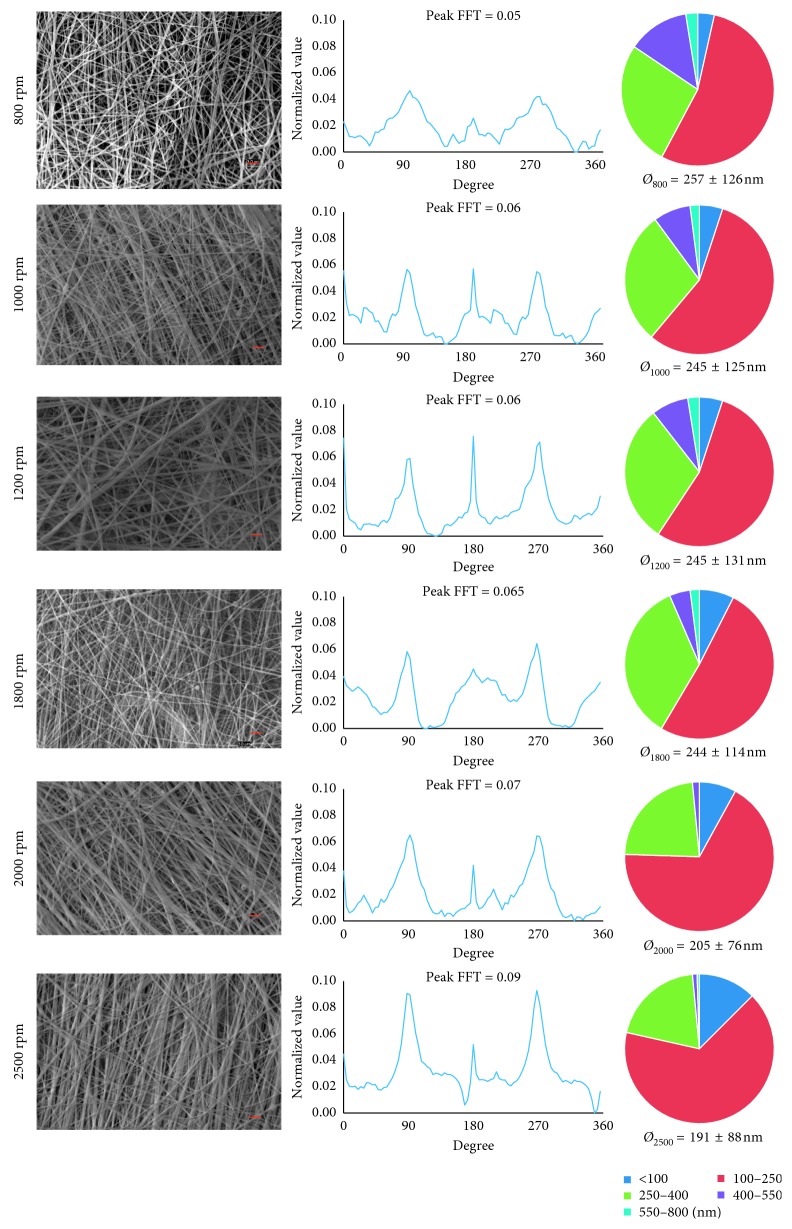
SEM micrographs of mat produced with the rotating drum collector at different rotation speeds (scale bar 2 *µ*m), 2D FFT analysis (peaks of alignment at 90° and 270°), fiber size distribution, and average diameters calculated using Image J software.

In order to generate mats with parallel-aligned nanofibers, the Cs/PEO blend was electrospun onto a rotating drum (Figure 1(c)). The drum rotation was varied from 800 rpm to 2500 rpm to analyze the influence of rotation speed on fiber alignment. Nanofiber mats morphology and mechanical properties have been evaluated by SEM and FTT, and by uniaxial tensile test, respectively.  Figure 5 shows SEM images of mats produced at all tested speeds of the rotating collector. All mats resulted bead-free and, by a preliminary qualitative observation of the images, it emerged that fibers aligned along the movement direction of the rotating drum, namely, in a parallel pattern, and that increasing the speed, fibers alignment increased. A more deep analysis of SEM images through Image J software put in evidence that an increase in the rotation speed leads to a decrease of the average fibers diameter (from 257 to 191 nm) and to an increase in the amount of fibers with diameter in a smaller range of 100–250 nm.

The FFT analysis of SEM images was used to quantitatively analyze the alignment degree of Cs nanofibers within the mats. As shown previously, the electrospinning of Cs/PEO blend solution onto a static plate resulted in a random nanofibrous mesh with no particular fiber orientation (Figure 4). On the contrary, increasing the speed of the rotating drum onto which the fibers were deposited resulted in an increasing fibers alignment, with an almost complete alignment achieved at the highest speeds examined (Figure 5). Two sharp peaks of alignment at a distance around 180° in the 2D FFT analysis and their increasing value (from 0.05 to 0.09) confirmed that increasing the rotation speed, a higher amount of fibers aligns along a preferential direction. Particularly, starting from 1800 rpm up to 2500 rpm, the values of the 2D FFT alignment peaks were >0.065, demonstrating the possibility to obtain significant fiber alignment using that range of rotation speed.

All typologies of the produced electrospun mats, i.e., at different speed rotations and cut out along different fiber orientations (parallel // and perpendicular orientation ⊥) (Figure 1(c)), underwent tensile test until failure.

**Figure 6:**
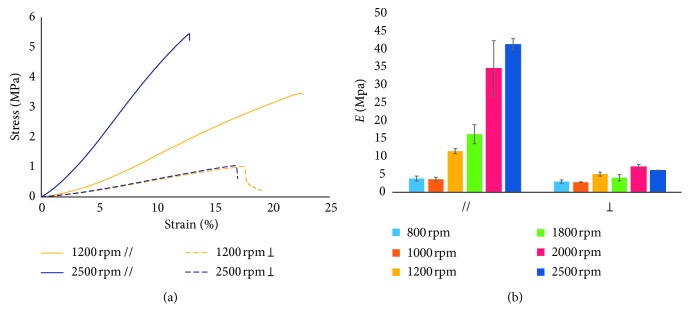
Mechanical tests on mats produced with the rotating drum collector. (a) Stress-strain curves of 1200 and 2500 rpm samples and (b) results of tensile tests at different rotation speeds and fiber orientations.

The results of the mechanical characterization are reported in Figure 6. From the stress-strain curves (Figure 6(a)), it is immediately noticeable that mats fabricated at the highest rotation velocity and tested along the nanofiber direction are stiffer and more resistant. Indeed, for specimens cut parallel to the rotation direction, increasing the rotation speed and consequently the degree of fibers alignment, a significant increase in tensile modulus (from 3.83 MPa to 41.32 MPa) and in stress at break (from 1.18 MPa to 7.77 MPa) was observed (Figures 6(a) and 6(b)). Interestingly, scaffolds produced at the same speeds but excised perpendicular to the rotation direction showed a considerably lower tensile modulus and stress at break (Figures 6(a) and 6(b) and Table 2), with values similar to those measured on random matrices produced using a static plate collector that do not significantly increase increasing the rotating velocity (from 2.98 MPa to 6.09 MPa). Conversely, the elongation at break does not significantly change either increasing the rotating velocity or according the testing direction, i.e., // or ⊥.

This anisotropy becomes less pronounced at lower speeds, particularly at drum rotation of 800 and 1000 rpm, where there is no significant difference between parallel and perpendicular specimens because of poor fibers alignment, similarly to what happens in random matrix. On the contrary, increasing speed rotation from 1200 to 2500 rpm, the fiber alignment in the direction of movement increases and so also the tensile strength and the stress at break in parallel direction. Therefore, in the parallel direction, the tensile modulus and stress at failure increase with speed rotation, except for the two lowest speeds of 800 and 1000 rpm.

### 3.4. Chitosan Circumferential-Orientated Nanofiber Mats: Rotating Disk Collector

**Figure 7:**
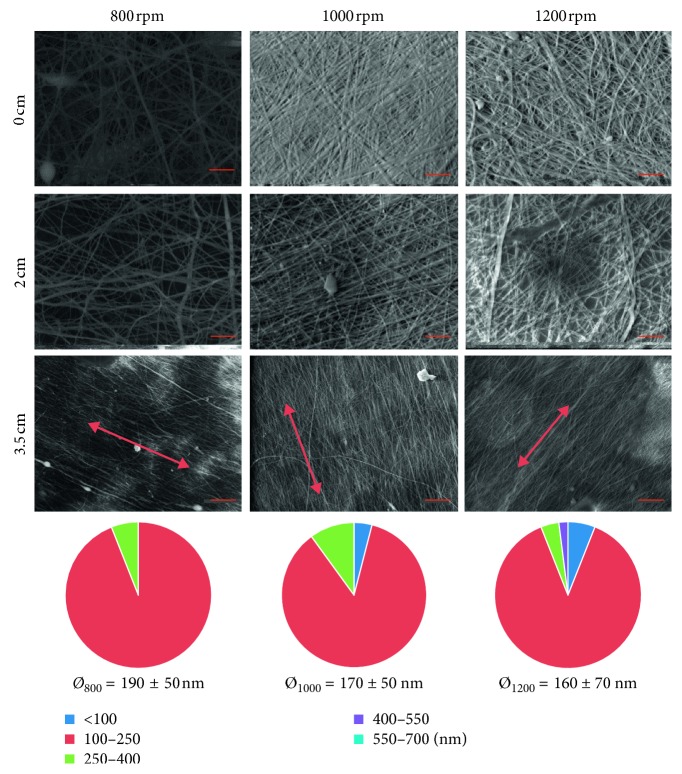
SEM micrographs of mats produced with rotating disk collector at 800, 1000, and 1200 rpm cut at 0, 2, and 3.5 cm from the center (scale bar 3 *µ*m), and fiber size distribution and average fiber diameters of 3.5 cm specimens.

In this study, for the first time, Cs/PEO nanofibers were electrospun onto a rotating disk collector, with the aim of obtaining circumferentially aligned (CircAl) Cs/PEO nanofibers. To generate fiber alignment, the spinneret was directed perpendicularly to a circular plate rotating from 800 rpm to 1200 rpm. In this configuration, the direction of movement is along the circumferential direction of the rotating circular surface. Fisher et al. [30] developed a mathematical model that provides an indication of circumferential poly(*ε*-caprolactone) (PCL) fiber deposition onto a rotating disk mandrel. The distribution of linear velocities on the disk collector depends on the distance from the center and can be calculated according to the equation of tangential velocity in uniform circular motion:

(2) $$\begin{array}{c} v=\frac{2\pi r}{T}n, \end{array}$$

where *r*, *n*, and *T* are, respectively, the disk radius, the rpm, and the period, i.e., the time a point takes to complete a turn. For example, for a disk with a radius of 5 cm, the tangential velocity *v* at the disk edge is about 6 m/s, while near the center, it is about 1 m/s. In their work, Fisher et al. simulated the distribution of PCL fibers deposited on the disk and assessed that higher rotational speed (i.e., higher rpm values) allows higher circumferential fibers deposition, while at lower speed, the jet instability produces random fibers orientation. Moreover, in the disk center, the fibers are orientated in random way, while at edge disk, they become aligned. Considering the above aspects, the obtained mats were cut at a distance of 0, 2, and 3.5 cm from the center, and then observed to SEM to calculate the fiber diameter and size distribution and estimate the alignment. SEM images (Figure 7) confirmed the mathematical model results, since near the center the fibers appear randomly aligned, while, gradually moving away from the center, an increase in fiber alignment can be observed, more evident at higher rpm. 3.5 cm from the center specimens at 800, 1000, and 1200 rpm show a decrease in the average fiber diameter and an increase in the number of fibers between 100 and 250 nm, increasing the rotation speed.

**Figure 8:**
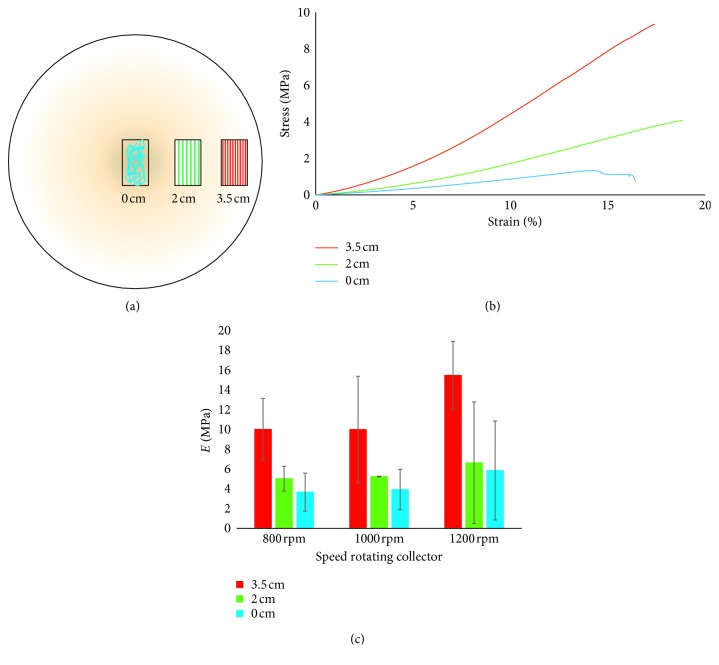
Mechanical test on mats produced with the disk-rotating collector. (a) Schematization of samples cut at 0, 2, and 3.5 cm from the center; (b) stress-strain curve of 1200 rpm samples; (c) results of tensile tests at different speeds rotations.

For the three different rotational speeds, samples at 0, 2, and 3.5 cm from the center were cut as in Figure 8(a) and tested to evaluate the mechanical properties. Tensile tests (Figures 8(b) and 8(c) and Table 3) show that for all rotation speeds, there is an increase in Young's modulus increasing the distance from the center.

This result can be ascribed to an increase in the fiber alignment approaching the mat edge. Considering Young's modulus, for each single distance from the center at different speed rotations, no significant differences can be observed, suggesting that the analyzed rotational speeds confer more or less the same degree of alignment without influencing significantly the mechanical properties. Looking at the stress at break values, it can be noticed (i) a slight increase increasing the distance from the center of the disk, although the differences are not significant, (ii) no differences increasing the velocity at the same distance from the disk center, hence confirming Young's modulus trend. Conversely, the elongation at break, at each single distance from the center, presents a tendency of increasing as the rotation speed increases, even if the differences are not significant. In general, all the three analyzed mechanical parameters (Young's modulus, stress, and elongation at break) of the membrane fabricated using the disk collector presented high standard deviation, suggesting a certain variability in the membrane structure.

From the comparison of the mechanical parameters obtained with the different used collector typologies, it can be put in evidence that Young's modulus and the stress at break of the 3.5 cm disk specimens at 1200 rpm are similar to the values obtained with the drum collector at 1800 rpm (parallel orientation). Therefore, 1200 rpm disk rotation is enough to induce fiber alignment and, consequently, improve the membrane mechanical properties. Conversely, the mechanical properties of the membranes obtained with (i) the static collector, (ii) drum collector at low speed, and (iii) the disk in the central sections that have a random distribution of the fibers are therefore comparable each other.

## 4. Discussion

The need of tissue engineering to produce scaffolds that mimic the aligned fibers present in native tissue and potentially guide the deposition of new ECM structures led to the development of electrospun Cs based mats with different fiber orientations, with the intent to create specifically oriented patterns able to guide tissue formation. To achieve this purpose, chitosan-based nanofiber membranes were fabricated through electrospinning. Particularly, three different collectors were tested: a static plate, a rotating drum, and a rotating disk, to generate, respectively, random, parallel, circumferential-oriented fibers. Chitosan was chosen for its interesting biological properties that make it a promising biomaterial for regenerative medicine applications. Although Cs has been electrospun before [21, 23], in this work, its spinnability in a circumferential configuration has been tested for the first time.

This study confirmed that the degree of fiber anisotropy can be tuned by using different collector geometries and also controlling the collector rotation velocity. This fibrous architecture in turn strongly affects scaffold mechanical properties. The comparison of the properties of random matrices produced with static plate or low speed rotation (800–1000 rpm) and those of the orientated mats (drum collector 1200–2500 rpm) showed a clear correlation between the obtained organized nanostructure and its mechanical behavior. Additionally, for the parallel aligned mats (1200–2500 rpm) fabricated using the rotating drum collector, Young's modulus and the stress at break in the fibers direction were significantly higher than those perpendicular to fibers direction one, underlining the anisotropic mechanical behavior of these aligned fibers mats. This characteristic is similar to tendon or ligaments tissue, which are stronger in the fibers direction and softer in the transverse one. Although these mats mimic tendon and ligaments anisotropy, they still do not have the same mechanical strength of native tissues [31, 32]. However, these mats are intended not to replace tendons and ligaments and therefore are not required to possess the same mechanical characteristics of such tissues but to lead the host cells to synthesize and deposit new matrix and hence to induce tissue regeneration. For this purpose, the values of the analyzed mechanical parameters have been evaluated mainly as key indicators of tissue architecture anisotropy. The obtained mechanical results are in accord to literature in which it has been reported that the fiber alignment has a profound effect on the anisotropic mechanical properties of [33] that vary significantly depending on the testing direction [34]. The average values of the mechanical parameters we obtained by using drum collector are not easily comparable to those reported by other authors with chitosan. In fact, it is worthy to point out, that the experimental conditions (i.e., chitosan concentration, crosslinking, rotating drum speed, etc.) reported in the other studies, though similar, are not identical to ours and therefore a rigorous comparison is not completely appropriated. Dey Sarkar et al. [5], for example, using a drum rotating speed of 200 rpm got significantly higher mechanical properties (stress at break of 5.2 ± 0.3 MPa and Young's modulus of 75.8 ± 10.2 MPa) than ours at 100 rpm (stress at break 0.18 ± 0.04 MPa and Young's modulus 1.52 ± 0.29 MPa). However, in that study, the membranes have been crosslinked and, especially, the mechanical tests were performed on dried samples. Also in [35], the higher mechanical properties measured for the uncrosslinked membranes are likely due to the dried condition used.

As already underlined, in this study for the first time, novel Cs nanofibrous mats with circumferentially orientation similar to native knee meniscus were successfully fabricated. To this aim, a rotating disk collector was used, in which fibers were oriented along the circumferential direction of the disc-shaped collecting mandrel.

From a mechanical point of view, however, the three analyzed mechanical parameters (Young's modulus, stress, and elongation at break) of the membranes fabricated using the disk collector presented high standard deviations, suggesting a certain variability in the membranes structure. This high variability could be due to the higher vibration we could observe during the electrospinning process onto the disk in comparison to drum. Further studies will be addressed to ameliorate the process, limit this variability, and obtain more reproducible results.

The obtained results, to the best of our knowledge, are the first ones reporting the fabrication of circumferential-oriented chitosan mats and indicate that electrospinning onto a rotating plate may provide an easy method for the fabrication of an anatomically inspired meniscus construct with direction-dependent mechanical properties.

## 5. Conclusions

In conclusions, chitosan nanofiber-oriented mats were successfully fabricated by electrospinning a Cs/PEO blend solution. Different fiber alignments were obtained by using different types of collector. A static plate allowed for producing nanofiber mats with random orientation, while parallelly oriented and circumferentially aligned nanofiber mats were obtained through a rotating drum and a rotating disk, respectively. Morphological and mechanical properties of all typologies of mats were evaluated in terms of alignment degree, average fibers diameter, diameters size distribution, and tensile properties. The use of a high-speed rotating drum collector led to a reduction in nanofiber diameter, an increase in fibers linear alignment, and an improvement in mechanical properties. Instead, the use of a disk collector induced the production of Cs nanofibrous mats with circumferential orientation similarly to native knee meniscus.

Further works are required for both one-direction aligned and circumferentially oriented fibers to improve scaffold architecture and mechanics. Multilayer hybrid structures can be produced in which, respectively, the chitosan electrospun mats overlap one another or are directly deposited on previously fabricate 3D freeze-dried scaffold. Crosslinking treatments can be, moreover, easily performed in order to further increase the mechanical properties of the chitosan nanofiber mats.

## Figures and Tables

**Table 1:** Processing parameters for Cs electrospinning.

Collector	Voltage (kV)	Flux (mL/min)	Distance tip to collector (cm)	Speed rotation (rpm)
Static plate	20–22	0.02	13	–

Drum collector	20–22	0.02	13	800
				1000
				1200
				1800
				2000
				2500

Disk collector	20–22	0.02	13	800
				1000
				1200

**Table 2:** Young's modulus (*E*), stress at break (*σ*_at break_), and elongation at break (*ε*_at break_) of the two different fibers orientations (// and ⊥) of Cs electrospun mats produced with rotating drum collector.

	//			⊥		
	*E* (MPa)	*σ*_at break_ (MPa)	*ε*_at break_ (%)	*E* (MPa)	*σ*_at break_ (MPa)	*ε*_at break_ (%)
800 rpm	3.83 ± 0.70	1.18 ± 0.21	17.16 ± 2.60	2.98 ± 0.46	0.63± 0.11	14.62 ± 1.55
1000 rpm	3.65 ± 0.54	0.83 ± 0.17	13.02 ± 0.70	2.81 ± 0.096	0.35 ± 0.13	9.95 ± 2.63
1200 rpm	11.48 ± 0.71	3.37 ± 0.59	22.47 ± 4.11	5.08 ± 0.55	1.08 ± 0.17	17.43 ± 0.74
1800 rpm	16.24 ± 2.67	3.91 ± 0.58	20.62 ± 2.98	4.07 ± 0.88	0.88 ± 0.09	16.28 ± 2.24
2000 rpm	34.61 ± 7.75	6.54 ± 1.92	16.94 ± 2.85	7.12 ± 0.69	1.42 ± 0.24	16.54 ± 2.21
2500 rpm	41.32 ± 1.64	7.77 ± 1.65	17.83 ± 3.87	6.09 ± 0.098	1.16 ± 0.23	16.78 ± 2.03

**Table 3:** Young's modulus (*E*), stress at break (*σ*_at break_), and elongation at break (*ε*_at break_) of different fiber orientations of Cs electrospun mats produced with rotating disk collector.

	Distance from center								
	0 cm			2 cm			3.5 cm		
	*E* (MPa)	*σ*_at break_ (Mpa)	*ε*_at break_ (%)	*E* (MPa)	*σ*_at break_ (Mpa)	*ε*_at break_ (%)	*E* (MPa)	*σ*_at break_ (Mpa)	*ε*_at break_ (%)
800 rpm	3.67 ± 1.92	2.92 ± 3.08	25.78 ± 12.56	5.03 ± 1.25	4.40 ± 3.70	28.44 ± 7.71	10.01 ± 3.12	2.54 ± 1.23	14.81 ± 4.83
1000 rpm	3.93 ± 2.04	1.16 ± 0.54	19.12 ± 10.14	5.24 ± 0.036	2.44 ± 1.33	22.89 ± 12.03	10.00 ± 5.38	3.21 ± 1.93	15.25 ± 5.07
1200 rpm	5.85 ± 5.01	1.48 ± 1.40	14.94 ± 4.34	6.63 ± 6.15	3.29 ± 3.92	17.01 ± 6.79	15.47 ± 3.44	4.88 ± 4.40	15.87 ± 5.59

## Data Availability

The data used to support the findings of this study are included within the article. If needed, further data can be requested to the corresponding author.
